# INDIVIDUALS AND THEIR CAREGIVERS WITH PERSISTENT PAIN: A FEASIBILITY AND ACCEPTABILITY STUDY

**DOI:** 10.1093/geroni/igac059.414

**Published:** 2022-12-20

**Authors:** Christine Fruhauf, Arlene Schmid, Aimee Fox, Jennifer Dickman Portz, Julia Sharp, Heather Leach, Marieke Van Puymbroeck

**Affiliations:** Colorado State University, Fort Collins, Colorado, United States; Colorado State University, Fort Collins, Colorado, United States; Kansas State University, Manhattan, Kansas, United States; University of Colorado Anschutz, Aurora, Colorado, United States; Colorado State University, Fort Collins, Colorado, United States; Colorado State University, Fort Collins, Colorado, United States; Clemson University, Clemson, South Carolina, United States

## Abstract

Persistent pain interventions targeting the caregiving dyad (i.e., caregivers and care receivers) are scarce. Thus, the purpose of this pilot study was to assess the feasibility and acceptability of the Merging Yoga and self-management to develop Skills (MY-Skills) intervention for caregiving dyads experiencing persistent pain. MY-Skills is a group intervention and was delivered in-person or online (due to COVID-19) twice a week for eight weeks, with each two-hour session including self-management education followed by yoga. Benchmarks for feasibility were set a prioi and included: recruitment, attrition, attendance, safety, acceptability/satisfaction, and study completion. Thirteen participants (caregivers n=7, care-receivers n=6) completed the in-person intervention and 18 individuals (9 dyads) completed the online version. Feasibility benchmarks were met, except for recruitment, where >1000 individuals were screened for eligibility. Interventions may lead to improved wellbeing, yet further research is needed to establish efficacy of health-related outcomes for the caregiving dyad experiencing persistent pain.

